# Preserving Biodiversity in Marginal Rural Areas: Assessment of Morphological and Genetic Variability of a Sicilian Common Bean Germplasm Collection

**DOI:** 10.3390/plants9080989

**Published:** 2020-08-04

**Authors:** Maria Carola Fiore, Francesco Maria Raimondo, Francesco Mercati, Ignazio Digangi, Francesco Sunseri, Anna Scialabba

**Affiliations:** 1 Council for Agricultural Research and Economics Research Centre for Plant Protection and Certification (S.S. 113 km 245,500), 90011 Bagheria, Italy; 2 PLANTA/Autonomous Center for Research, Documentation and Training, 90123 Palermo, Italy; francesco.raimondo@unipa.it; 3 Institute of Biosciences and Bioresources (IBBR), National Research Council, Corso Calatafimi 414, 90129 Palermo, Italy; francesco.mercati@ibbr.cnr.it; 4 Living Plants Germplasm Bank of Nebrodi, Contrada Pirato, 98060 Ucria (ME), Italy; ignazio.digangi62@gmail.com; 5 Department of Agraria, University Mediterranea of Reggio Calabria, Località Feo di Vito snc, 89124 Reggio Calabria, Italy; francesco.sunseri@unirc.it; 6 Department of Biological, Chemical and Pharmaceutical Science and Technologies (STEBICEF), University of Palermo, Via Archirafi 38, 90123 Palermo, Italy; anna.scialabba@unipa.it

**Keywords:** common bean, genetic diversity, morphological descriptors, seedbank

## Abstract

The historical cultivation of common bean (*Phaseolus vulgaris* L.) has resulted in the development of local populations/cultivars in restricted Italian rural areas. Many common bean landraces, still cultivated in small mountain areas from Sicily, have become outdated and endangered due to the commercial varieties spreading. These accessions are poorly known but often represent a genetic heritage to be preserved and enhanced. The ex situ conservation of fifty-seven Sicilian common bean landraces was carried out at the “Living Plants Germplasm Bank” at Ucria (Messina, Italy), founded by the Nebrodi Regional Park, together with the “Sicilian Plant Germplasm Repository” of University of Palermo (SPGR/PA). To assess the germplasm genetic diversity, nineteen morphological traits and eight Simple Sequence Repeats (SSRs) were used. Genetic distances among landraces were calculated to construct a clustering tree by using unweighted pair group method arithmetic (UPGMA). Seed germplasm diversity of Sicilian common bean varied from 80.7% to 93.3%, based on six seed descriptors and six leaf, flower, and pod descriptors, respectively, while cluster genetic analysis depicted a clear separation among all the 57 landraces. Principal coordinates (PCoA) and STRUCTURE analyses showed a prevalent rate of admixture between Mesoamerican and Andean gene pools in Sicilian common bean collection, confirming its heterogeneity. The observed high level of diversity evidenced the needs to adopt accurate criterion to plan a definitive ex situ germplasm collection to share agrobiodiversity with local farmers and to avoid any further loss of genetic resources in rural and protected areas.

## 1. Introduction

Common bean (*Phaseolus vulgaris* L.) is a major grain legume consumed worldwide as important source of proteins, minerals (mostly zinc and iron) and vitamins for human diets [1], resulting one of the most important food crops due to high revenues for producers and its ecological diversity. It is native to the New World and classified in two major gene pools, namely Mesoamerican and Andean, firstly based on morphological traits [2], phaseolin seed proteins [3,4], and allozymes [5,6]. More recently, molecular markers spanning broader genomic areas as AFLP [7,8], chloroplast [9,10], and microsatellites [11,12,13] confirmed these two distinct gene pools. The domestication process took place after their formation, causing marked changes at morphological and genetic level [14,15,16,17,18]. In Europe, common bean landraces exhibit inter- and intra-population diversity [17] making the Old Continent a potential secondary center of diversification for this legume.

The introduction of common bean into cultivation in Italy from America through Spain has been found in a detailed historical framework, but there are reliable lines of evidence of its presence by the early 16th century [18]. Nowadays, common bean is the major cultivated legume in Italy, with a dry beans production of 11,981 tons and over 6411 cultivated hectares [19]. Despite the widespread adoption of modern varieties, many farmers preserved traditional common bean landraces. They have local names due to seed color and cultivation area, showing specific traits well adapted to local environmental conditions, often characterized by higher nutritional properties and/or particular tastes very appreciated by consumers [18,20]. Landraces from many Italian regions have been developed over several centuries of cultivation and are often highly variable at the phenotypic level. In this respect, Sicily offers several local varieties still cultivated under different environmental conditions and many of them are under extinction risk.

In agricultural cropping systems, the interaction between environment and anthropogenic impact contributes significantly to intraspecific variability. Further, changes in environmental conditions had enormous impact on crop diversity, and the replacement of landraces with few high-yielding modern varieties caused deep genetic erosion, resulting in loss of specific genes and complexes, as well as recombination. Altogether, these events contributed to intraspecific genetic variability, changing allele frequencies and therefore crop population structure [21,22]. Different mating systems may also influence the amount of genetic diversity within and between populations [23], thus constituting an important parameter in developing strategies for germplasm conservation. Allelic richness is a measure of genetic diversity indicative of a population’s long-term potential for adaptability and persistence [24,25].

Thus, germplasm conservation plays an important role in counteracting constant threats to biodiversity, in maintaining a healthy ecosystem, global agriculture and food safety. Biobanks engage in preserving genetic resources and distributing biospecimens with their associated data, characterized by high complexity, for research purposes [26]. This process has increased the need to work with high quality, non-redundant specimens with a wide genetic diversity. Ex situ plant germplasm conservation aims to preserve the genetic resources derived from cultivated and wild forms, as seeds, tissues, cells, embryos, pollen, and DNA, for long-term storage under suitable conditions.

The management of ex situ seed viability in genebanks is a key element to maintain plant genetic resources [27] and is challenging mainly because of these collections size. The Global Plan of Action for the Conservation and Sustainable Utilization of Plant Genetic Resources for Food and Agriculture [28], recommended the increase of the efficiency in ex situ conservation of specimens by reducing duplicate accessions within and between the collections, while preserving as much as possible the overall genetic diversity. The selected accessions define a core collection [29,30] and contains as much genetic diversity as possible.

In detail, redundancy should be kept below 10% of the total collection, should contain at least 70% of the variation in the whole collection [31]. Once the entries for the core collection have been identified, important decisions are necessary to be made regarding storage, the frequency of accessions regeneration, the managing of germplasm information and associated data electronically. The establishment of the core collection based on phenotypic traits results not enough to evaluate germplasm diversity, requiring also a genetic analysis approach. For this purpose, molecular markers such as microsatellites (simple sequence repeats, SSRs) have also been successfully used to assess intra- and interspecific genetic diversity of common bean to define germplasm collections and to assist the conservation of important repositories throughout the world [32,33,34,35,36,37].

Ex situ local crop conservation in genebanks and in situ conservation on farms can both curb biodiversity loss in rural and protected areas and maintain socio-ecological system resilience [38]. Both approaches allow us to long-term preserve representative collection (ex situ), as well as to maintain the accession genetic variation in response to environmental conditions changes and crop management (in situ) [39].

The “Regional Park of Nebrodi” is one of the largest protect area in Sicily (860 km^2^) which together with Madonie Mountains form the Sicilian portion of the Apennine mountain chain characterized by rural small-scale traditional communities, typical of the Mediterranean basin. Established on 1993, the “Regional Park of Nebrodi” is engaged in wild biodiversity and agrobiodiversity preservation highly interconnected with local peasant agriculture. The Sicilian Plant Germplasm Repository at the University of Palermo (SPGR/PA), together with the “Living Plant Germplasm Bank” (LPGB) of Ucria (ME) [40] currently carry out their research activities devoted to the conservation and exploitation of common bean landraces. The LPGB houses ex situ germplasm collections are located inside the Nebrodi Mountains area for seed multiplication and storage. The SPGR/PA, *Hortus Botanicus Panormitanus* seed bank from 1993 [41], collects, preserves, enhances, and redistributes endangered species, wild crop progenitors and cultivated species of Mediterranean areas and can be considered as a driving force for biodiversity conservation [42].

The aim of this study was firstly the assessment of genetic diversity of common bean germplasm collection at SPGR/PA, mainly representative of Nebrodi Mountains area. Further, the data analysis was used to define a core collection throughout morphological and genetic characterization.

## 2. Results

### 2.1. Morphological Diversity

**Figure 1:**
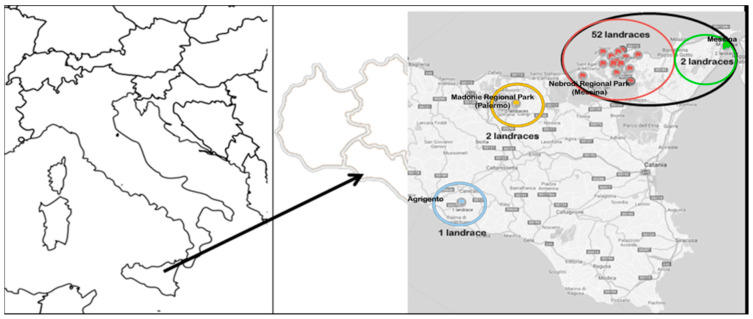
Map of Sicily. Colored circles indicate the different macro areas where the common bean germplasm was collected.

A total of eighteen morphologic traits, twelve qualitative and six quantitative, were used to evaluate 57 common bean landraces from the germplasm collections at SPGR/PA (Table S1) collected from different geographic Sicilian areas (Italy) (Figure 1).

**Figure 2:**
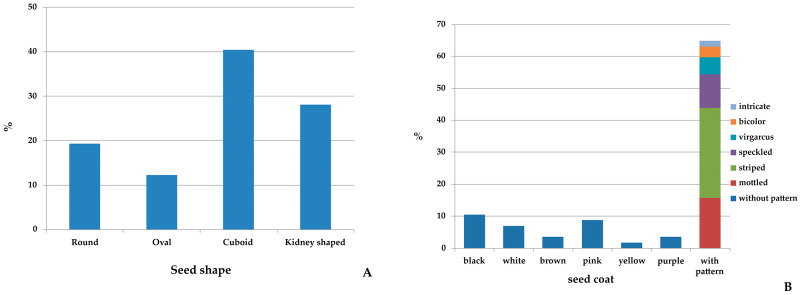
Percentage (%) of seed shape (**A**) and seed coat color and pattern (**B**) recorded on 57 Sicilian common bean landraces. In panel B the different types of seed coat pattern are reported.

**Figure 3:**
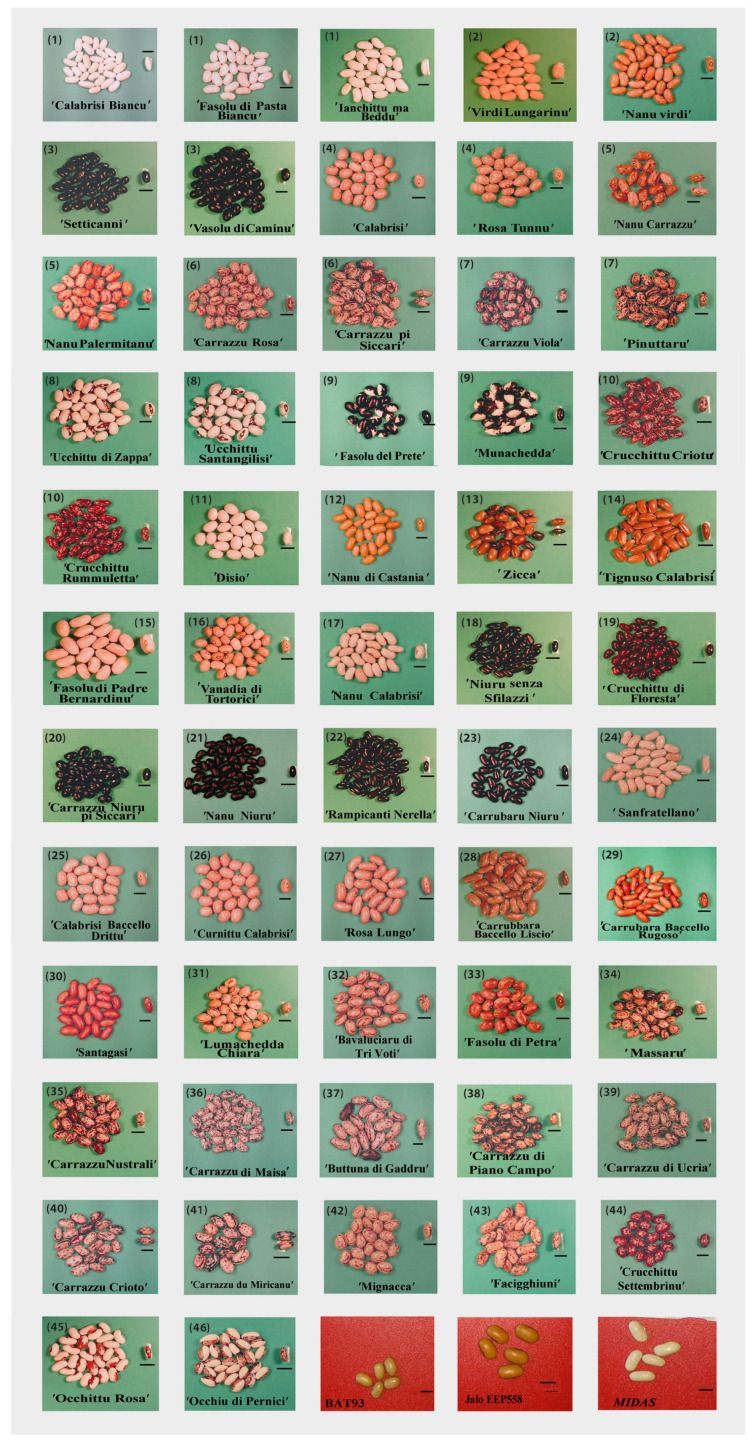
Fifteen-seven Sicilian common bean landraces conserved at SPGR/PA and “Living Plants Germplasm Bank” at Ucria sorted by seed morphotypes reported in Figure S2. Number in brackets = group morphotype. Three standard common bean genotypes are included (red background). Bar = 1 cm.

The seed, pod, flower, and leaf descriptors are reported in Table S1. The most spread seed shape in the collection was cuboid (40.3%), followed by kidney (28.0%), round (19.3%) and oval (12.3%) (Figure 2A). Moreover, a wide variation for seed colors and their patterns was observed, many common bean landraces (65%) showed seed coat with colors pattern, while the remaining harbored black, white, brown, pink, yellow and purple color (Figure 2B). The background color of seed coat pattern was usually pinkish (59%) with red or purple as predominant secondary color (Table S1). Striped was the pattern mainly distributed among the landraces (28.1%) followed by mottled (15.8), speckled (10.5%), virgarcus (5.3%), bicolor (3.5%), and intricate (1.7%) (Figure 2B). Two landraces (‘Fasolu del Prete’ and ‘Munachedda’) showed a similar pattern bicolor (black and white), whereas another three (‘Ucchittu di Zappa’, ‘Ucchittu Santangiulisi’ and ‘Occhittu Rosa’) showed a virgarcus seed coat pattern type around hilum, and finally a landrace (‘Occhiu di Pirnici’) showed an intricate pattern with striped around hilum and white as prevalent seed coat color (Figure 3).

Three landraces (‘Carrazzu Crioto’, ‘Carrazzu du Miricanu’ and ‘Carrazzu pi Siccari’) showed a different color of strips each, while one (‘Nanu Carrazzu’) harbored different seed coat color background (Figure S1). To check for a potential gene flow in these four landraces, percentage of seed with different coat color were counted in a 50 seeds bulk for two generations (2016–2017). Segregation for the seed trait was not observed in the self-progeny, as the ratio of different coat color between generations (Table S2).

Seed morphology on the whole collection (Figure 3) described 46 morphotypes identified with a code (Figure S2). Three landraces (‘Calabrisi Bianco’, ‘Fasolu di Pasta Bianco’ and ‘Ianchittu ma Beddu’) showed the same seed-morphotype with code 001043 (morphotype 1), characterized by the absence of colors pattern, showing white seed coat color, kidney shape and seed dull (Figure 3). Nine other morphotypes (005045, 080015, 145315, 265135, 265137, 275135, 365235, 671123, and 981315) included two landraces each (from morphotype 2 to 10) (Figure 3). The remaining 36 landraces exhibited unique morphotype each (Figure 3 and Figure S2). With the addition of six flower, pod, and leaf descriptors into the analysis, fifty-six different morphotypes were recognizable, while only two landraces, ’Ucchittu di Zappa’ and ’Ucchittu Santangilisi’, confirmed the same code (671123). The predominant flower color among landraces was white (26%) followed by lilac (24%). One landrace (’Munachedda’) showed yellow flower, despite shares the same seed-morphotype of ’Fasolu del Prete’ that instead harbors white flowers (Table S1).

Descriptive analysis of quantitative traits among all landraces are reported in Table S3. According to the coefficient of variation (CV), the common bean germplasm displayed wide phenotypic variation for dry bean yield (47%) and low variation for seed length (17%). Large seeds (100-seed weight above 40 g) represent over the 90% of all the landraces (Figure S3) and four of five landraces with a 100-seed weight < 40g showed black seed coat color (Table S1).

All the data were subjected to analysis of variance (ANOVA) and the results revealed a significant effect (*p* < 0.001) for all variables (Table S4). The post-hoc Tukey’s test identified significant differences between pairs of means (Table S5). Mainly, the landrace ’Buttuna di Gaddru’ showed higher values on five out of seven variables, with a dry bean yield (2.55 kg m^−2^) not significant different by the mean value. The higher value for 100-seed weight was recorded by ‘Facigghuini’ (80.3 g) and the lowest by ‘Nanu Niuru’ (24.9 g), the latter was not significantly different from ‘Rampicante Nerella’ (25.1 g) and ’Carrabbaru Niuru’ (31.2 g). The post-hoc Tukey’s test on dry bean yield identified significant differences between landraces and the standard cultivar Borlotto. In particular, ’Ucchittu di Zappa’ (5.2 kg m^−2^) and ’Fasolu di Padre Bernardinu’ (4.56 kg m^−2^) showed a significantly higher yield than *cv*. Borlotto (3.79 kg m^−2^), while ’Nanu di Castania’ shows the lowest value of dry bean yield (0.43 kg m^−2^) despite a 100-seed weight (53.0g) close to mean value. Fourteen landraces showed a dry bean yield values not significantly different compared to *cv*. Borlotto (3.79 kg m^−2^) and 43% of landraces show a value above the mean (2.32 kg m^−2^) (Table S5).

**Figure 4:**
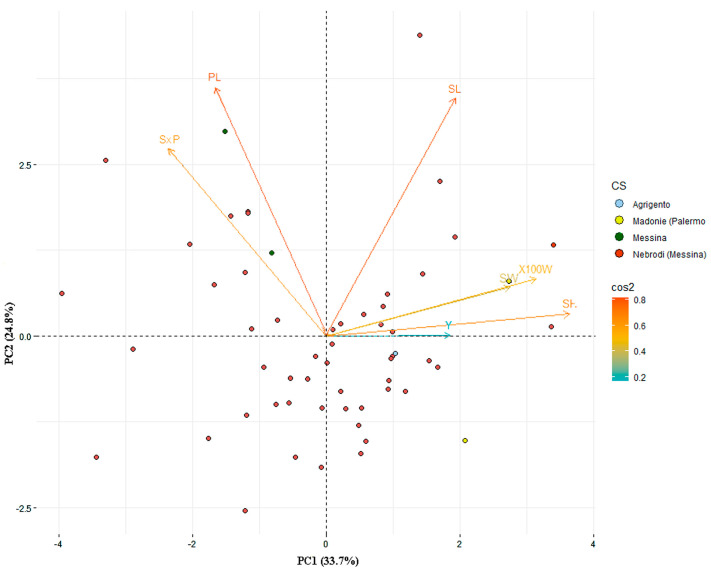
Principal component analysis (PCA) for quantitative parameters detected on 57 Sicilian common bean accessions. Based on their collection macro site (CS), samples were organized in four groups and associated traits to landraces separation were indicated by colored vector in the plot, underlining their significance values (0.2 < cos2 < 0.8). SL = seed length; SH = seed height; SW = seed width; S × P= seed/pod; PL = pod length; 100W = 100-seed weight; Y = dry bean yield.

**Figure 5:**
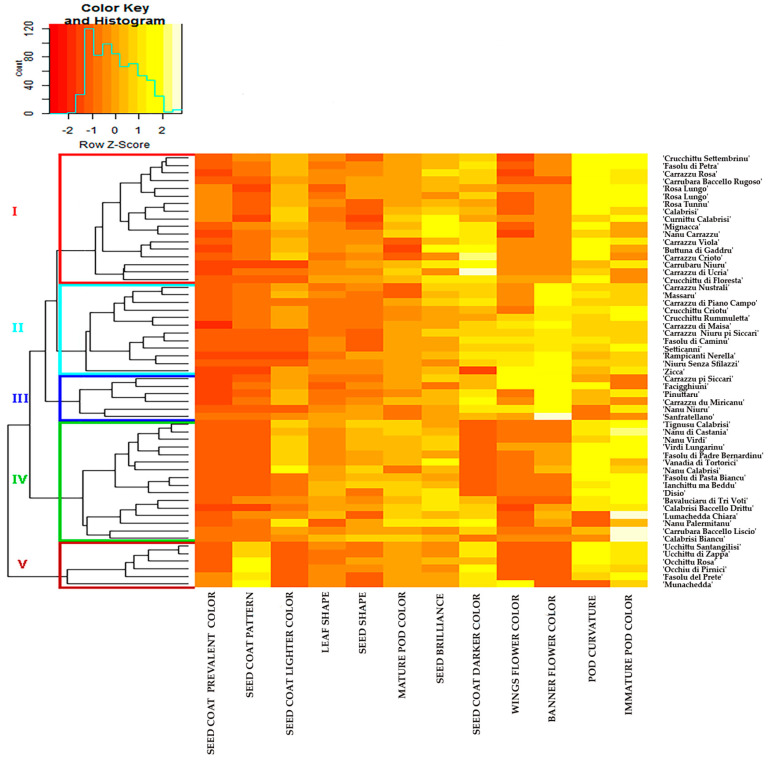
Heatmap of seed, pod and flower morphological traits of 57 Sicilian common bean accessions based on Euclidean distances. Red and yellow colors represent reduced and augmented representation levels, respectively. Hierarchical clustering of accessions (from cluster I to V) and traits are also shown.

Principal component analysis (PCA) displayed two principal components that contributed for 58.4% to overall variability among the Sicilian common bean landraces (Figure 4). A heatmap based on Euclidean distance and morphological measurements clustered the 57 landraces into five distinct groups (Figure 5), mainly based on geographical distribution (Table S6) and seed coat color. The first cluster (red line) enclosed 17 accessions, mainly collected into the area of Nebrodi Mountains and one (’Rosa Tunnu’) collected in a different area (Province of Agrigento). The second cluster (light blue) was mainly represented by accessions showing seed coat pattern with prevalent dark color (7), and black seed coat color (5). Six accessions, characterized by seed coat with darker colors, also belonged to cluster III (blue). By contrast, cluster IV (green) grouped 16 accessions showing light colors of seed coat without pattern, of which eleven were collected in areas closest to the plain and coast. The last cluster (darker red) gathered six accessions characterized by particular seed coat pattern, of which two (’Munachedda’ and ’Facigghiuni’) are collected in the area of Madonie Mountains.

### 2.2. Genetic Diversity

Eight SSR markers (PV-ag001, GATS91, BM159, BM160, BM172, BM210, PVBR25, and PVBR163), chosen among the most informative and useful for common bean genetic analyses, allowed us to record a total of 75 alleles and the number of alleles (allelic richness) per locus ranges from 5 (BM159) to 13 (PVBR25) as reported in Table 1. In detail, three loci (GATS91, BM160 and PVBR25) counted more than 10 alleles. The expected heterozygosity (He) ranged from 0.524 to 0.867 and four loci (GATS91, BM210, BM160, and PVBR25) showed higher values than the mean value 0.722.

The lowest polymorphic information content (PIC) was 0.463 (PV-ag001) and the highest 0.853 (BM160) and the detected value of power discrimination (PD) at all the loci (0.8656) indicated a high SSR efficiency (Table 1). BM160 was the most discriminant SSR (PD = 0.97) together with PVBR25 (PD = 0.94). The probability of identity (PI) was very low, ranging from 0.288 (PVag001) to 0.0318 (BM160). The combined estimate was 2 × 10^−8^, meaning that the probability of getting the same genotype in two accessions is almost null. The percentage of homozygosity (Hom) at each locus, as expected for an autogamous species, ranged from 56% (PVBR163) to 98% (BM210 and BM160).

Rare and private alleles within each accession were also evaluated (Table S7). The germplasm collection exhibited a significant number of rare alleles (36). Seventeen accessions showed a total of 21 private alleles with the landrace ’Occhiuttu Rosa’ carrying the greater number, three private alleles at three different loci (BM160, BM172 and BM210), followed by ’Carrazzu Criotu’ with two private alleles at one locus (PVBR163).

**Figure 6:**
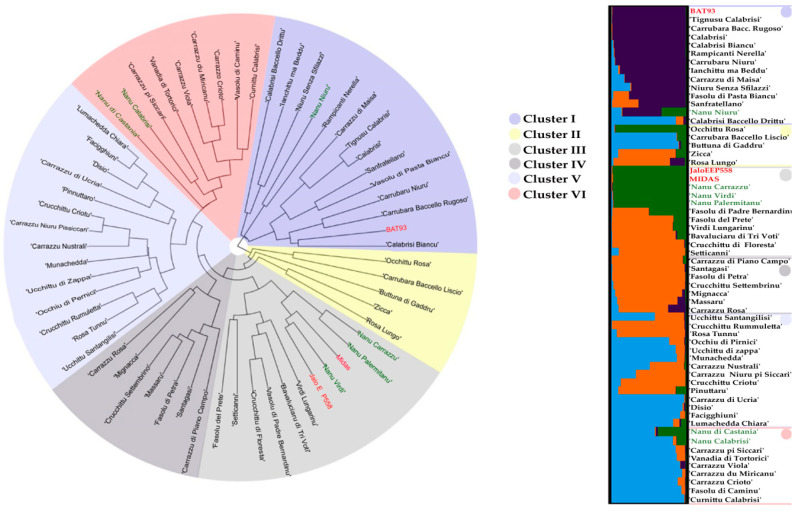
On the left phylogenetic analysis of Sicilian common bean germplasm collection, based on Bruvo’S distance coefficient and UPGMA cluster analysis. JaloEEP558, Midas (Andean) and BAT93 (Mesoamerican) were added as references. On the right Structure inferred for common bean germplasm collection analyzed. A horizontal line represents each sample. The length of colored segment highlights the membership percentage of samples to each group.

Samples belonging to the Sicilian common bean germplasm collection were grouped in six main clusters by UPGMA analysis based on Bruvo’s distance (Figure 6). In cluster I, twelve landraces characterized by the absence of seed coat pattern were included together with the Mesoamerican standard genotype (BAT93). Interestingly, this cluster contains all the landraces with seed morphotype 1. The two Andean references genotypes (JaloEEP558 and Midas) were included in cluster III together with three determinate landraces (’Nanu Palermitanu’, ’Nanu Carrazzu’, and ’Nanu Virdi’) and six indeterminate ones with or without seed coat pattern. Finally, the other determinate accessions were included in cluster VI. All the other indeterminate accessions, collected in different macro sites, clustered separately.

PCoA and STRUCTURE analysis are in agreement, grouping the germplasm in four groups/pools (Figure S4, Table S8). PCoA separated the Sicilian accessions in the four quadrants, placing Andean references and four determinate samples in the same area, while STRUCTURE analysis underlined as optimum number of genetic pools at K = 4 (Table S8), supporting the differences previously observed by genetic distance. Eight Sicilian common bean landraces belonging to pool 1 (violet) together with BAT93; pool 2 (green) was represented by Jalo EEP558, Midas, three out of five determinate landraces, and ’Occhittu Rosa’, the only one climbing accession. Finally, pool 3 (orange) appeared as a private Sicilian group with 15 accessions; while other 17 including ’Nano Calabrisi’ belonged to pool 4 (light blue). Based on the admixture coefficient (Q) ≥ 0.8 as the probability to assign each sample to a specific pool, 80% of samples has been assigned to a specific pool. By contrast, only 12 samples showed admixture genetic profile, of which 10 and 2 belonging to indeterminate and determinate Sicilian germplasm, respectively. The accessions do not appear assigned to different groups based on their geographic origin.

The Mantel test between genetic and morphological distance matrices showed a low negative (r: −0.019), albeit not significant (*p* > 0.05) correlation.

## 3. Discussion and Conclusions

In situ and ex-situ germplasm collections of crop species represent a valuable integrate strategy for landraces conservation for its exploitation by breeding programs. Plant breeders evaluate a large number of accessions looking for new sources for genetic improvement. The management of a germplasm collection needs to prioritize a limited number of accessions and, at the same time, to maximize available genetic diversity. Several common bean landraces are still cultivated in Sicily, mainly in smallholder-farmer systems and farmer-named cultivars [43] often derived on the redundancy in the germplasm collection [44].

Nineteen morpho-phenotypic descriptors [45,46] and eight SSRs, chosen among the most informative and useful to characterize different Italian [35,47,48,49] and worldwide collections [20,32,50,51,52], were used to assess the genetic diversity of a Sicilian common bean collection, avoiding its genetic erosion and to establish a core collection at the seed bank of SPGR/PA.

The results of the current study revealed different features of the 57 common bean accessions. Morphological seed traits characterization allowed us to identify 46 seed morphotypes of which thirty-six as unique profile. Common bean landraces belonging to the same seed morphotype were distinguished by SSR analysis. Thus, seed morphology could be considered a relevant tool to identify landraces or varieties as already reported in a large collection (4274 accessions) from 58 countries [52]. Moreover, three seed descriptors (height, length, and 100-seed weight) appeared most important for landraces distinctness in our collection by the principal component analysis (PCA), as previously reported [36,53].

Germplasm diversity of Sicilian common bean collection increased from 80.7% to 93.3%, adding six leaf, flower, and pod traits to seed descriptors. The high level of morphological variability across the 57 accessions is comparable to that observed in others Italian collection [48,54,55]. In detail, different patterns of diversity were detected and some of them showed specific and not spread pattern as ’Occhiu di Pernice’. Two landraces (’Fasolu del Prete’ and ’Munachedda’) collected in different macro sites (Nebrodi and Madonie Mountains, respectively) showed the same seed morphotype (981315; black and white bicolor pattern), similar to the local Sicilian variety named ’Badda’, included in the National list of traditional agri-food product and cultivated in a limited area of Madonie Mountains. Cluster analysis in the heatmap, defined by twelve variables, highlighted an appreciable distinctness between ’Fasolu del Prete’ and ’Munachedda’ due to flower, pod, and leaf descriptors and this was confirmed by UPGMA analysis, where the genetic distance appeared noticeable. Indeed, UPGMA analysis showed a closest clustering between ’Fasolu del Prete’ and one of the Andean reference genotypes (Jalo EEP558), while ’Munachedda’ clustered in a group with genetic admixture. These results are in agreement with a previous study [56], where a close relationship between ’Badda’ and the Andean gene pool (JaloEEP558) is reported using ISSR markers. Therefore, morphological traits allowed us to distinguish ’Munachedda’ and the Sicilian landrace ’Badda’, both cultivated in confining area of Madonie Mountains.

The heatmap based on Euclidean distance is mainly defined by two morphological descriptors (seed coat color and seed coat color pattern) and the different geographic collection site. Landraces collected in Nebrodi Mountains were included in all five morphological clusters as well as in all six clusters obtained by UPGMA analysis.

The genetic analysis of 57 Sicilian landraces showed a high degree of homozygosity, as expected by a self-pollinating species [57]. We detected an average of 9.4 alleles per locus, a higher value than those detected in a smaller germplasm collection (25 landraces) of Nebrodi Mountains obtained by using different SSR markers [54], but not so far from those reported in others Italian germplasm collections [48,49,55]. Moreover, our findings highlighted the importance of the detected high genetic diversity in Sicilian due to its mainly distribution in more geographically restricted area (860 km^2^) compared to those related to other Regional Italian collections (more than 15,000 km^2^). The efficiency of each SSRs in genetic fingerprinting was rather high for all the loci (mean 0.8656) and BM160 appeared the most discriminant locus. The PIC value of locus BM160 showed similar values to those detected in previous Italian germplasm studies [48,49,58], by contrast, lower PIC values were observed in worldwide collection [51,59]. The discriminating power for each locus was comparable or higher than that reported for the same SSR locus (GATS91, BM210, BM160, PVBR25, BM172, PVBR163) in previous Italian collection studies [47,48,49]. Furthermore, mean He across eight SSR loci was higher (0.70) in our collection compared to those observed in other studies carried out on Italian [9,35,47,48,49] as well as European collections [37,60,61,62]. The overall loci probability of identity (PI) and PIC values obtained in our study prove the ability of this SSR panel to distinguish among common bean landraces of Nebrodi Mountains. Different results were obtained in a smaller collection of common bean germplasm of the Nebrodi Mountains using different SSRs [54].

The presence of 36 rare alleles, 21 of which private, detected in about 58% of landraces underlined the high differentiation of our germplasm collection. High priority in SPGR/PA seed bank will be reserved to the landraces containing rare alleles, and even more private alleles, whose presence is an important feature for germplasm collection because proven to be informative for genetic conservation [63].

PCoA and STRUCTURE analysis indicated a large rate of admixture (61%) confirming the differentiated origin of the Sicilian common bean collection here reported. The germplasm spread from Andean and Mesoamerican gene pools in Europe aided the gene flow between them, by increasing the genetic diversity found in thousand landraces grown in small farms [64]. Our finding clearly showed the clustering of a larger number of accessions (23%) with the Mesoamerican gene pool than the Andean gene pool (16%), in contrast to those reported for Italian germplasm local/regional collections [9,17,18,48] and other European germplasm [61,62,65]. The absence of seed coat pattern in the accessions included in the Mesoamerican cluster appeared in agreement with a recent report [66]. Nine Sicilian landraces assigned to Mesoamerican gene pool showed a mean 100-seed weight of 43.1 g, while nine landraces assigned to Andean gene pool showed a mean 100-seed weight of 57.5 g. These findings are in agreement with its assignation to Andean gene pool, that generally contains large-seeded types [67]. Moreover, the Sicilian landraces clustered with BAT93, was mainly collected in flat land area, more closed to the coast, suggesting a better adaptive response to specific agro-environment conditions. Structure analysis also indicated that the determinate genotypes shared more Andean than Mesoamerican gene pool, in agreement with previous studies [5,68].

Not significant correlation (r = 0.01) was found between morphological and genetic distances by using the Mantel test. This could be due to the limited number of morphological traits and/or SSR loci analyzed.

Analysis of variance (ANOVA) indicated the high yield variability of the Sicilian common bean landraces when cultivated under the same pedoclimatic condition. Among the collection, 25% of the landraces showed a comparable yield performance to the standard modern cultivar ’Borlotto’ widely cultivated in Sicily. Two landraces, ’Ucchittu di Zappa’ and ’Fasolu di Padre Bernardinu’, showed a higher level of dry bean production.

The high genetic diversity detected in our Sicilian common bean germplasm highlight its potential economic importance for finding adaptive traits to stressful environments and low inputs condition frequently present in the marginal areas [69,70,71], where is actually confined the legume cultivation in Italy. In the area of Sicilian common bean cultivation local farmers probably carried out a selection of genotypes, firstly introduced during the Spanish domination (16–17th centuries) and maintained in a restricted area of cultivation, as evidenced by the local nomenclature. The conservation and sustainable use of farmers’ landraces needs more information on their adaptive, agronomic, and quality traits through also the support of biotechnology. The germplasm characterization can therefore capitalize what farmers have pursued for centuries through the development of a more sustainable agriculture. The large variability of pedoclimatic conditions in the marginal areas of Nebrodi Mountains allowed several generations of farmers to select common bean landraces hand down over the centuries.

Morphological and molecular techniques were able to detect high level of phenotypic and genetic diversity in the common bean collection from Sicily at SPGR/PA; these data set allowed us to detect some redundancies useful to define an ex situ core collection. Community-based conservation should be shared with local farmers, whose could directly benefit from this research to curb biodiversity loss and to maintain the genetic variation as evolution of the landrace itself in response to environmental changes in rural and protected areas. More interestingly, this study well reported the key role of the regional parks in conserving local agrobiodiversity for supporting a model to sustain the economy of local communities.

## 4. Materials and Methods

### 4.1. Plant Material

Fifty-seven household seed stock samples of common bean (*Phaseolus vulgaris* L.), consisting of fifty-one indeterminate climbing and six determinates, were collected from local growers in different geographical areas of Sicily (Italy) in 2014. In particular, fifty-four landraces were representative of Province of Messina including Regional Park of Nebrodi Mountains, two were collected from single farms located in Madonie Regional Park (Province of Palermo) and one from a farm of Province of Agrigento (Table S6, Figure 1).

To renew plant material in the SPGR/PA and to increase seed availability for request of local farmers, each accession was sown and grown for following growing seasons at the Experimental Station of “Living Plants Germplasm Bank” of Ucria (38°02’55" N 14°52’36" E, 850 m a.s.l.) every year. The germplasm collection is conserved at SPGR/PA, while the voucher specimens are housed at *Herbarium Mediterraneum* of the University of Palermo (PAL), which is useful for future reference.

Harvested seeds from each accession were divided in two lots: One assigned for long term storage in SPGR/PA, after dehydration, at low humidity and low temperature storage at −20 °C [72], the other one was used for seed multiplication. With the aim to evaluate crop productivity of Sicilian landraces compared to a variety “Borlotto-type”, wide cultivated in Sicily, a field trial was carried out at local farm (450 m a.s.l.), according to a randomized blocks experimental design with three replications, in 2015. The experimental unit was three rows 6 m long, with row and intra-row spacing 0.6 and 0.1 m, respectively. BAT 93, Jalo EEP558 and Midas genotypes were used as standard varieties for Mesoamerican (the first) and Andean (the others) gene pool in genetic characterization.

### 4.2. Morpho-Phenotypic Seed Analysis

Morphological traits assessment was carried out on five randomly plants for each landrace, according to International Board for Plant Genetic Resources (IBPGR) *Phaseolus vulgaris* L. descriptor list [45] and to Bioversity International/Centro Internacional de Agricultura Tropical (BI/CIAT) [46] (Table S9). Seed morphologic traits were recorded on 20 seeds for each landrace. In particular, six seed descriptors were analyzed: Coat pattern (SCP), color of coat darker (CSCD) and lighter (CSCL), prevalent color of coat (PCSC), shape (SSH) and brilliance (BS). Regarding the seed coat color, the term ‘prevalent’ was adopted by using 3 different states: (1) lighter color as background and darker color as stripes; (2) darker color as background and lighter color as stripes; (3) darker color and lighter color equally distributed [58]. Others six morpho-descriptors were added to the accession characterization: Color of flower banner (CFB) and wings (CFW), immature (IPC) and mature (MPC) pod color, pod curvature (PC) and leaf shape (LS). Seven quantitative variables were also included: Seed length (SL), seed height (SH), seed width (SW), number of seed per pod (S × P), pod length (PL), 100 seed weight (100 W), and dry bean production (Y).

### 4.3. DNA Isolation and Amplification

Young leaves for each landrace, collected from plants grown at LPGB of Ucria, were freeze-dried and stored at the Tissue Bank of SPRGR/PA. Powered material was utilized for DNA extraction by using NucleoSpinPlant II kit (Macherey-Nagel), according to manufacture procedures. DNA quality and quantity were analyzed using a Thermo Scientific™ NanoDrop 2000c spectrophotometer.

Eight SSR loci (Table S10) were selected from previous genetic studies based on their Polymorphic Information Content (PIC) and dispersed map locations [50,73,74]. The eight SSR primer pairs were multiplexed, labelling their forward primer with ATTO565, HEX, or FAM (Eurofin Genomics), respectively (Table S10). PCR amplification was performed in a 25 µL final volume containing 12.5 µL of My Taq TM HS Mix (BIOLINE), 0.5 µL of primers (20 µM each) and 20 ng of DNA, using the following touchdown PCR program: 5 min initial denaturation step at 95 °C, followed by 10 cycles of 94 °C for 30 s, 60 s annealing at 65 °C (with 1 °C decrease per cycle), 60 s extension at 72 °C. Products were subsequently amplified for 25 cycles of 30 s denaturation at 94 °C, 60 s annealing at 55 °C and 60 s extension at 72 °C, with a final extension for 10 min. The fragments were analyzed on ABI3730 DNA Analyzer (Applied Biosystem) sequencing machine.

### 4.4. Data Analyses

Quantitative variables were analyzed using one-way ANOVA and mean comparison with the Tuckey-b test by using R package multcomp. Quantitative and qualitative variables were normalized during the analysis to balance the influence of each set of variables.

Principal component analysis (PCA) was performed to define the most determinant quantitative variables able to discriminate among accessions by using the R packages FactoMiner [75] and factoextra (https://cran.r-project.org/package=factoextra). A cluster analysis, based on Euclidean distances, for qualitative morpho-phenotypic traits, was performed on all the investigated accessions and UPGMA (unweighted pair group method with arithmetic mean) tree was developed using the function heatmap.2 in R package gplots (https://github.com/talgalili/gplots).

Genetic diversity per locus was evaluated through several parameters, such as number of alleles per locus (Na), number of rare and private alleles [76], observed heterozygosity (Ho), expected heterozygosity value (He), probability of identity (PI), polymorphic information content (PIC), percentage of homozygosity (Hom), and power discrimination (PD) by using GENEALEX 6.502 [77] and CERVUS program version 3.0.7 [78].

Genetic relationships among samples were investigated through UPGMA cluster analysis. Phylogenetic tree was developed by using R package poppr [79], performing the bootstrap analysis with 1000 re-samplings and using Bruvo distance [80]. The K-means algorithm (find.clusters) to independently verify the samples assignment to each cluster was used. Genetic similarities between genotypes were also determined using a principal coordinate analysis (PCoA) by adegenet [81]. Finally, to further evaluate the population structure of collection studied a Bayesian clustering was performed by STRUCTURE software [82], as reported in Mercati et al. [83]. An ad hoc statistic [84] was adopted to highlight the most probable K value, to reduce a possible overestimation of subgroup number by STRUCTURE. Samples with membership probabilities ≥ 0.80 were assigned to the corresponding subgroup.

To study the relationships between genetic and Euclidean distance of samples, the Mantel test [85] was also carried out using R/ecodist v2.0.1 package [86].

## Figures and Tables

**Table 1:** Descriptive statistics of genetic variation for each locus. Number of alleles (Na), Observed heterozygosity (Ho), Expected heterozygosity (He), Polymorphic Information Content (PIC), Probability of Identity (PI), Percentage of homozygosity (Hom), Power discrimination (PD).

Locus	Na	Range Size (bp)	Ho	He	PIC	PI	Hom	PD
**PVag001**	6	137–155	0.333	0.524	0.463	0.288	66.7	0.7120
**GATS91**	11	214–256	0.035	0.733	0.709	0.0916	96.5	0.9084
**BM210**	9	167–195	0.0350	0.746	0.713	0.0972	98.2	0.9028
**BM160**	12	180–256	0.019	0.867	0.853	0.0318	98.2	0.9682
**BM159**	5	187–199	0.123	0.656	0.593	0.181	91.2	0.8189
**PVBR25**	13	149–177	0.182	0.802	0.785	0.0564	82.5	0.9436
**BM172**	9	70–112	0.089	0.636	0.590	0.1687	94.7	0.8313
**PVBR163**	10	221–259	0.439	0.646	0.607	0.1606	56.1	0.8394
**Overall**	9.375 ^a^	-	0.157 ^a^	0.701 ^a^	0.664 ^a^	2 × 10^−8 b^	85.5 ^a^	0.8656 ^a^

^a^ Average of the estimated value across all loci. ^b^ Combined non-exclusion probability (identity), considering all loci.

## Supplementary Materials

The following are available online at https://www.mdpi.com/2223-7747/9/8/989/s1, Figure S1: Four Sicilian common bean landraces sampled in local farm with different seed coat color. The segregating progeny were checked in order to exclude seed mixtures or gene flow. Bar = 1 cm, Figure S2 Graphic representation of relative frequency distribution of 46 seed group morphotypes, within the 57 common bean landraces collected in different areas of Sicily, based on six seed descriptors. In red group number of morphotype also reported in Figure 3, Figure S3: Frequency distribution of six classes of 100-seed weight (g) within the 57 *Phaseolus vulgaris* L. landraces, Figure S4: Relationships between determinate and indeterminate Sicilian accessions and Andean (Jalo EEP558, Midas) and Mesoamerica (BAT93) references as represented by the first two principal coordinates of a Principal Coordinates Analysis (PCoA) using allelic profiles from 8 SSR molecular markers, Table S1: Quanti-qualitative descriptors of seed, flower, leaf and pod recorded on fifty-seven *Phaseolus vulgaris* L. landraces conserved at SPGR/PA, Table S2: Percentage of seed with different striped seed coat color recorded on four landraces across three years, Table S3: Descriptive statistic of quantitative variables detected on 57 Sicilian landraces. SH = seed height; SW = seed width; SL = seed length; PL = pod length; S × P = seed/pod; 100W = 100-seed weight; Y = dry bean yield; SD = Standard Deviation, CV = Coefficient of Variation expressed as a percentage, Table S4: Analysis of variance (ANOVA) of quantitative parameters, SH = seed height; SW = seed width; SL = seed length; PL = pod length; S × P = seed/pod; 100W = 100-seed weight; Y = dry bean yield, Table S5: Means comparison and post hoc test (Tukey’s) for quantitative parameters. SH = seed height; SW = seed width; SL = seed length; PL = pod length; SxP= seed/pod; 100W = 100-seed weight; Y = dry bean yield. Only dry bean yield (Y) parameter was compared to Borlotto type as control, Table S6. Accession number, local name, collection site (CS), geographic coordinates sites (GCS) and macro sites of sampled landraces, Table S7: Rare alleles detected on landraces in homozygous or heterozygous state, Table S8: Posterior membership coefficients following a STRUCTURE analysis and K = 4, Table S9: List of BI/CIAT descriptors (2009) and code number used for morphological characterization of Sicilian common bean germplasm collection. Numbers in parentheses, under each descriptor, corresponding IPGRI descriptor numbers (1982), Table S10: Microsatellite primer pairs (SSR) used for genetic characterization of Sicilian common bean germplasm collection.

## References

[1] Beebe S., Gonzalez A.V., Rengifo J. (2000). Research on trace minerals in the common bean. Food Nutr. Bull..

[2] Gepts P., Debouck D., van Schoonhoven A., Voysest O. (1991). Origin, domestication, and evolution of the common bean (*Phaseolus vulgaris* L.). Common Beans: Research for Crop Improvement.

[3] Gepts P., Bliss F.A. (1986). Phaseolin variability among wild and cultivated common beans (*Phaseolus vulgaris*) from Colombia. Econ. Bot..

[4] Gepts P., Bliss F.A. (1988). Dissemination pathways of common bean (*Phaseolus vulgaris*, Fabaceae) deduced from phaseolin electrophoretic variability. II. Europe and Africa. Econ. Bot..

[5] Singh S.P., Nodari R., Gepts P. (1991). Genetic diversity in cultivated common bean: I. allozymes. Crop. Sci..

[6] Debouck D.G., Toro O., Paredes O.M., Johnson W.C., Gepts P. (1993). Genetic diversity and ecological distribution of *Phaseolus vulgaris* (Fabaceae) in northwestern South America. Econ. Bot..

[7] Svetleva D., Pereira G., Carlier J., Cabrita L., Leitao J., Genchev D. (2006). Molecular characterization of *Phaseolus vulgaris* L. genotypes included in Bulgarian collection by ISSR and AFLP™ analyses. Sci. Hortic..

[8] Šustar-Vozlič J., Maras M., Javornik B., Meglič V. (2006). Genetic diversity and origin of slovene common bean (*Phaseolus vulgaris* L.) germplasm as revealed by AFLP markers and phaseolin analysis. J. Am. Soc. Hortic. Sci..

[9] Sicard D., Nanni L., Porfiri O., Bulfon D., Papa R. (2005). Genetic diversity of *Phaseolus vulgaris* L. and *P. coccineus* L. landraces in central Italy. Plant Breed..

[10] Desiderio F., Bitocchi E., Bellucci E., Rau D., Rodriguez M., Attene G., Papa R., Nanni L. (2013). Chloroplast microsatellite diversity in *Phaseolus vulgaris*. Front. Plant Sci..

[11] Blair M.W., Giraldo M.C., Buendía H.F., Tovar E., Duque M.C., Beebe S. (2006). Microsatellite marker diversity in common bean (*Phaseolus vulgaris* L.). Appl Genet..

[12] Kwak M., Gepts P. (2009). Structure of genetic diversity in the two major gene pools of common bean (*Phaseolus vulgaris* L., Fabaceae). Theor. Appl. Genet..

[13] Blair M.W., Díaz J.M., Hidalgo R., Diaz L.M., Duque M.C. (2007). Microsatellite characterization of Andean races of common bean (*Phaseolus vulgaris* L.). Theor. Appl. Genet..

[14] Papa R., Nanni L., Sicard D., Rau D., Motley T.J., Zerega N., Cross H. (2006). The evolution of genetic diversity in *Phaseolus vulgaris* L.. Darwin’s Harvest: New Approaches to the Origins; Evolution and Conservation of Crops.

[15] Papa R., Bellucci E., Rossi M., Leonardi S., Rau D., Gepts P., Nanni L., Attene G. (2007). Tagging the Signatures of Domestication in Common Bean (*Phaseolus vulgaris*) by Means of Pooled DNA Samples. Ann. Bot..

[16] Bitocchi E., Bellucci E., Giardini A., Rau D., Rodriguez M., Biagetti E., Santilocchi R., Spagnoletti Zeuli P., Gioia T., Logozzo G. (2013). Molecular analysis of the parallel domestication of the common bean (*Phaseolus vulgaris*) in Mesoamerica and the Andes. New Phytol..

[17] Angioi S.A., Rau D., Attene G., Nanni L., Bellucci E., Logozzo G., Negri V., Spagnoletti Zeuli P.L., Papa R. (2010). Beans in Europe: Origin and structure of the European landraces of *Phaseolus vulgaris* L.. Theor. Appl. Genet..

[18] Piergiovanni A.R., Lioi L. (2010). Italian common bean landraces: History, genetic diversity and seed quality. Diversity.

[19] Food and Agriculture Organization of the United Nations. http://www.fao.org/faostat/en/#home.

[20] Blair M.W., González L.F., Kimani P.M., Butare L. (2010). Genetic diversity, inter-gene pool introgression and nutritional quality of common beans (*Phaseolus vulgaris* L.) from Central Africa. Theor. Appl. Genet..

[21] Loveless M.D., Hamrick J.L. (1984). Ecological determinants of genetic structure in plant populations. Annu. Rev. Ecol. Syst..

[22] Linhart Y.B., Grant M.C. (1996). Evolutionary significance of local genetic differentiation in plants. Annu. Rev. Ecol. Syst..

[23] Schoen D.J., Brown A.H. (1991). Intraspecific variation in population gene diversity and effective population size correlates with the mating system in plants. Proc. Natl. Acad. Sci. USA.

[24] Hurlbert S.H. (1971). The nonconcept of species diversity: A critique and alternative parameters. Ecology.

[25] Petit R.J., El Mousadik A., Pons O. (1998). Identifying populations for conservation on the basis of genetic markers. Conserv. Biol..

[26] de Souza Y.G., Greenspan J.S. (2013). Biobanking past; present and future: Responsibilities and benefits. AIDS.

[27] van Treuren R., de Groot E.C., van Hintum T.J.L. (2013). Preservation of seed viability during 25 years of storage under standard genebank conditions. Genet. Resour. Crop Evol..

[28] FAO Global Plan of Action for the Conservation and Sustainable Utilization of Plant Genetic Resources for Food and Agriculture and the Leipzig Declaration. Proceedings of the International Technical Conference on Plant Genetic Resources.

[29] Beckman H.B., Franckel R.M. (1984). The Effect of Physician Behavior on the Collection of Data. Ann. Intern. Med..

[30] Brown A.H.D. (1989). Core collections: A practical approach to genetic resources management. Genome.

[31] van Hintum T.J.L., Brown A.H.D., Spillane C. (2000). Core Collections of Plant Genetic Resources.

[32] Díaz L.M., Blair M.W. (2006). Race structure within the Mesoamerican gene pool of common bean (*Phaseolus vulgaris* L.) as determined by microsatellite markers. Theor. Appl. Genet..

[33] Logozzo G., Donnoli R., Macaluso L., Papa R., Knüpffer H., Spagnoletti Zeuli P.L. (2007). Analysis of the contribution of Mesoamerican and Andean gene pools to European common bean (*Phaseolus vulgaris* L.) germplasm and strategies to establish a core collection. Genet. Resour. Crop Evol..

[34] Blair M.W., Díaz L.M., Buendía H.F., Duque M.C. (2009). Genetic diversity, seed size associations and population structure of a core collection of common beans (*Phaseolus vulgaris* L.). Theor. Appl. Genet..

[35] Angioi S.A., Rau D., Rodriguez M., Logozzo G., Desiderio F., Papa R., Attene G. (2009). Nuclear and chloroplast microsatellite diversity in *Phaseolus vulgaris* L. from Sardinia (Italy). Mol. Breed..

[36] Perseguini J.M.K.G., Silva G.M.B., Rosa J.R.B.F., Gazaffi R., Marçal J.F., Carbonell S.A.M., Chiorat A.F., Zucchi M.I., Garcia A.A.F., Benchimol-Reis V. (2015). Developing a common bean core collection suitable for association mapping studies. Genet. Mol. Biol..

[37] Leitão S.T., Dinis M., Veloso M.M., Šatović Z., Vaz Patto M.C. (2017). Establishing the bases for introducing the unexplored portuguese common bean germplasm into the breeding world. Front. Plant Sci..

[38] Bacchi M., Leone M., Mercati F., Preiti G., Sunseri F., Monti M. (2010). Agronomic evaluation and genetic characterization of different accessions in lentil (*Lens culinaris* Medik.). Ital. J. Agron..

[39] Gómez-Baggethun E., Corbera E., Reyes-García V. (2013). Traditional ecological knowledge and global environmental change: Research findings and policy implications. Ecol. Soc..

[40] Scialabba A., Bartolotta I., Digangi I., Geraci M., Raimondo F.M., Spadaro V. (2016). The Botanical Garden “Bernardino da Ucria” in the Natural Park of the Nebrodi (Sicily) and its mission to conserve, exploit and spread local agrobiodiversity and officinal plants. III International Plant Science Conference (IPSC)-111 Congresso Società Botanica Italiana.

[41] Scialabba A., Raimondo F.M. (1994). La banca del germoplasma dell’Orto Botanico dell’Università di Palermo: Prime esperienze. Inform. Bot. Ital..

[42] Scialabba A., Raimondo F.M. (2019). The “Sicilian Plant Germplasm Repository” of the University of Palermo: 25 years of activity in biological conservation. Bocconea.

[43] Harlan J.R. (1975). Our vanishing genetic. Science.

[44] van Treuren R., van Hintum T.J. (2003). Marker-assisted reduction of redundancy in germplasm collections: Genetic and economic aspects. Acta Hortic..

[45] (1982). International Board for Plant Genetic Resources (IBPGR/IPGRI) Biodiversity International. Phaseolus Vulgaris Descriptors.

[46] Biodiversity International, Rome (Italy/Centro Internacional de Agricultura Tropical (BI/CIAT) (2009). Key Access and Utilization Descriptors for Bean Genetic Resources. https://www.bioversityinternational.org/.

[47] de Luca D., Cennamo P., del Guacchio E., di Novella R., Caputo P. (2018). Conservation and genetic characterization of common bean landraces from Cilento region (southern Italy): High differentiation in spite of low genetic diversity. Genetica.

[48] Mercati F., Leone M., Lupini A., Sorgona A., Bacchi M., Abenavoli M.R., Sunseri F. (2013). Genetic diversity and population structure of a common bean (*Phaseolus vulgaris* L.) collection from Calabria (Italy). Genet. Resour. Crop. Evol..

[49] Scarano D., Rubio F., Ruiz J.J., Rao R., Giandomenico C. (2014). Morphological and genetic diversity among and within common bean (*Phaseolus vulgaris* L.) landraces from the Campania region (Southern Italy). Sci. Hortic..

[50] Buso G.S.C., Amaral Z.P.S., Brondani R.P.V., Ferreira M.E. (2006). Microsatellite markers for the common bean *Phaseolus vulgaris*. Mol. Ecol. Notes.

[51] Burle M.L., Fonseca J.R., Kami J.A., Gepts P. (2010). Microsatellite diversity and genetic structure among common bean (*Phaseolus vulgaris* L.) landraces in Brazil; a secondary center of diversity. Theor. Appl. Genet.

[52] Rana J.C., Sharma T.R., Tyagi R.K., Chahota R.K., Gautam N.K., Mohar S., Sharma P.N., Ojha S.N. (2015). Characterisation of 4274 accessions of common bean (*Phaseolus vulgaris* L.) germplasm conserved in the Indian gene bank for phenological, morphological and agricultural traits. Euphytica.

[53] Chiorato A.F., Carbonell S.A.M., Benchimol L.L., Chiavegato M.B., Dias L.A.S., Colombo C.A. (2007). Genetic diversity in common bean accessions evaluated by means of morpho-agronomical and RAPD data. Sci. Agric..

[54] Lioi L., Nuzzi A., Campion B., Piergiovanni A. (2012). Assessment of genetic variation in common bean (*Phaseolus vulgaris* L.) from Nebrodi mountains (Sicily, Italy). Genet. Resour. Crop Evol..

[55] Raggi L., Tiranti B., Negri V. (2013). Italian common bean landraces: Diversity and population structure. Genet. Resour. Crop Evol..

[56] Paniconi G., Gianfilippi F., Mosconi P., Mazzucato A. (2010). Distinctiveness of bean landraces in Italy: The case study of the ‘Badda’ bean. Diversity.

[57] Bradshaw J.E. (2016). Genetic structure of landraces. Plant Breeding: Past, Present and Future.

[58] Carucci F., Garramone R., Aversano R., Carputo D. (2017). SSR markers distinguish traditional Italian bean (*Phaseolus vulgaris* L.) landraces from Lamon. Czech J. Genet. Plant Breed..

[59] Pipan B., Meglič V. (2019). Diversification and genetic structure of the western-to-eastern progression of European *Phaseolus vulgaris* L. germplasm. BMC Plant Biol..

[60] Sarıkamış G., Yaşar F., Bakır M., Kazan K., Ergül A. (2009). Genetic characterization of green bean (*Phaseolus vulgaris*) genotypes from eastern Turkey. Genet. Mol. Res..

[61] Carović-Stanko K., Liber Z., Vidak M., Barešić A., Grdiša M., Lazarević B., Šatović Z. (2017). Genetic Diversity of Croatian Common Bean Landraces. Front. Plant Sci..

[62] Maras M., Pipan B., Sustar-Vozlic J., Todorović V., Đurić G., Vasić M., Kratovalieva S., Ibusoska A., Agić R., Matotan Z. (2015). Examination of genetic diversity of common bean from the Western Balkans. J. Am. Soc. Hort. Sci..

[63] Kalinowski S.T. (2004). Counting alleles with rarefaction: Private alleles and hierarchical sampling designs. Conserv. Genet..

[64] Gioia T., Logozzo G., Marzario S., Spagnoletti Zeuli P.L., Gepts P. (2019). Evolution of SSR diversity from wild types to U.S. advanced cultivars in the Andean and Mesoamerican domestications of common bean (*Phaseolus vulgaris*). PLoS ONE.

[65] Bitocchi E., Rau D., Bellucci E., Rodriguez M., Murgia M.L., Gioia T., Santo D., Nanni L., Attene G., Papa R. (2017). Beans (*Phaseolus* ssp.) as a Model for Understanding Crop Evolution. Front. Plant Sci..

[66] Caproni L., Raggi L., Ceccarelli S., Negri V., Carboni A. (2019). In-depth characterisation of common bean diversity discloses its breeding potential for sustainable agriculture. Sustainability.

[67] Gepts P., Gepts P. (1988). A Middle American and an Andean Common Bean Gene Pool. Genetic Resources of Phaseolus Beans. Current Plant Science and Biotechnology in Agriculture.

[68] Kwak M., Toro O., Debouck D.G., Gepts P. (2012). Multiple origins of the determinate growth habit in domesticated common bean (*Phaseolus vulgaris*). Ann. Bot..

[69] Casquero P.A., Lema M., Santalla M., de Ron A.M. (2006). Performance of Common Bean (*Phaseolus vulgaris* L.) Landraces from Spain in the Atlantic and Mediterranean Environments. Genet. Resour. Crop. Evol..

[70] Assefa T., Wu J., Beebe S.E., Rao J.M., Marcomin D., Claude R.J. (2015). Improving adaptation to drought stress in small red common bean: Phenotypic differences and predicted genotypic effects on grain yield, yield components and harvest index. Euphytica.

[71] Darkwa K., Ambachewa D., Mohammed H., Asfawa A., Blair M.W. (2016). Evaluation of common bean (*Phaseolus vulgaris* L.) genotypes for drought stress adaptation in Ethiopia. Crop. J..

[72] Scialabba A., Raimondo F.M. (2012). *Hortus Botanicus Panormitanus* seed bank. Studi Trent. Sci. Nat..

[73] Yu K., Park S.J., Poysa V., Gepts P. (2000). Integration of simple sequence repeat (SSR) markers into a molecular linkage map of common bean (*Phaseolus vulgaris* L.). J. Hered..

[74] Gaitán-Solís E., Duque M.C., Edwards K.J., Tohme J. (2002). Microsatellite repeats in common bean (*Phaseolus vulgaris* L.): Isolation, characterization, and cross-species amplification in *Phaseolus* ssp.. Crop. Sci..

[75] Le S., Josse J., Husson F. (2008). FactoMineR: An R Package for Multivariate Analysis. J. Stat. Softw..

[76] Marshal D.R., Brown A.H.D., Frankel O.H., Hawkes J.G. (1975). Optimum sampling strategies in genetic conservation. Crop Genetic Resources for Today and Tomorrow.

[77] Peakall R., Smouse P.E. (2006). GenAlEx 6: Genetic analysis in Excel. Population genetic software for teaching and research. Mol. Ecol. Notes.

[78] Kalinowski S.T., Taper M.L., Marshall T.C. (2007). Revising how the computer program CERVUS accommodates genotyping error increases success in paternity assignment. Mol. Ecol..

[79] Kamvar Z.N., Tabima J.F., Grünwald N.J. (2014). Poppr: An R package for genetic analysis of populations with clonal, partially clonal, and/or sexual reproduction. PeerJ.

[80] Bruvo R., Michiels N.K., D’souza T.G., Schulenburg H. (2004). A simple method for the calculation of microsatellite genotype distances irrespective of ploidy level. Mol. Ecol..

[81] Jombart T., Ahmed I. (2011). Adegenet 1.3-1: New tools for the analysis of genome-wide SNP data. Bioinformatics.

[82] Pritchard J.K., Stephens M., Donnelly P. (2000). Inference of population structure using multilocus genotype data. Genetics.

[83] Mercati F., Longo C., Poma D., Araniti F., Lupini A., Mammano M.M., Fiore M.C., Abenavoli M.R., Sunseri F. (2015). Genetic variation of an Italian long shelf-life tomato (*Solanum lycopersicon* L.) collection by using SSR and morphological fruit traits. Genet. Resour. Crop. Evol..

[84] Evanno G., Regnaut S., Goudet J. (2005). Detecting the number of clusters of individuals using the software structure: A simulation study. Mol. Ecol..

[85] Mantel N. (1967). The detection of disease clustering and a generalized regression approach. Cancer Res..

[86] Goosle S.C., Urban D.L. (2007). The ecodist Package for Dissimilarity-based Analysis of Ecological Data. J. Stat. Softw..

