# Supplementary material for: Preserving Biodiversity in Marginal Rural Areas: Assessment of Morphological and Genetic Variability of a Sicilian Common Bean Germplasm Collection
Source: Plants (Basel). 2020 Aug 4;9(8):989. doi: 10.3390/plants9080989 (PMC7463873; doi:10.3390/plants9080989)
Supplement: Supplementary file 1 [file plants-09-00989-s001.zip › plants-858461 - supplemenary figures for XML.pdf]

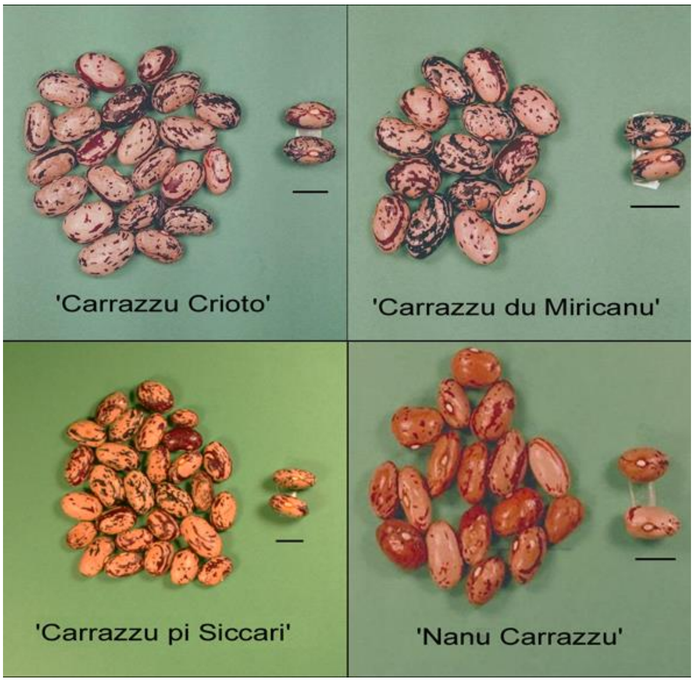

Figure S1. Four Sicilian common bean landraces sampled in local farm with different seed coat color. The segregating progeny were checked in order to exclude seed mixtures or gene flow. Bar = 1 cm**.**

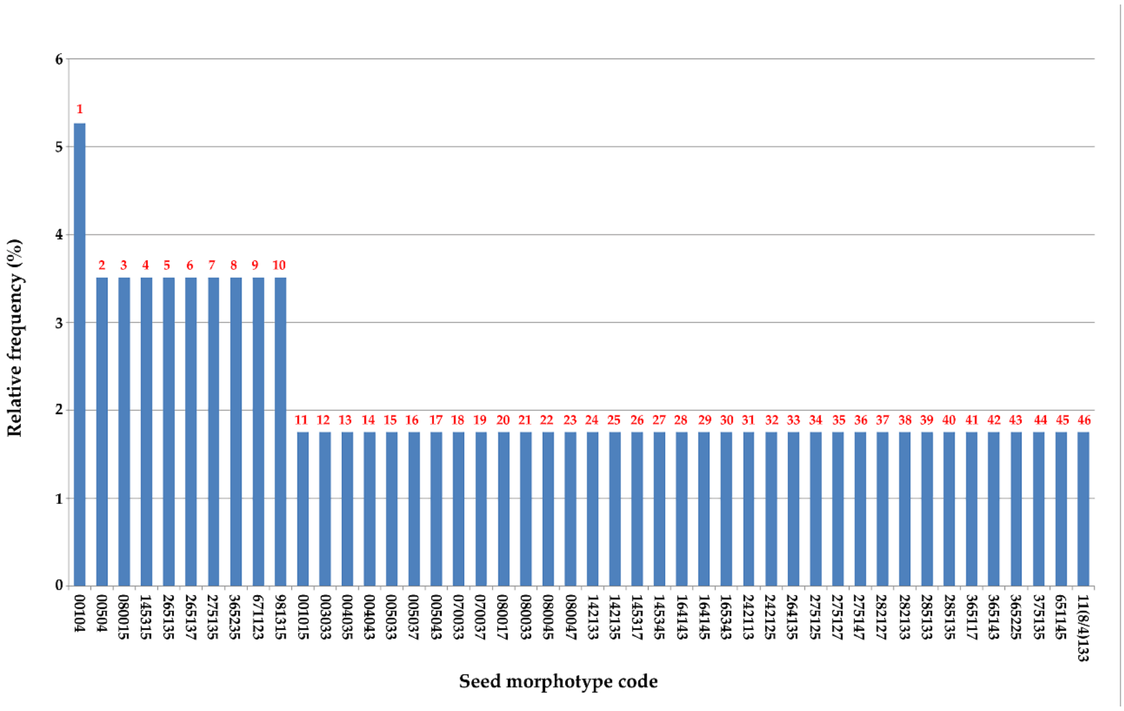

Figure S2. Graphic representation of relative frequency distribution of 46 seed group morphotypes, within the 57 common bean landraces collected in different areas of Sicily, based on six seed descriptors. In red group number of morphotype also reported in Figure 3.

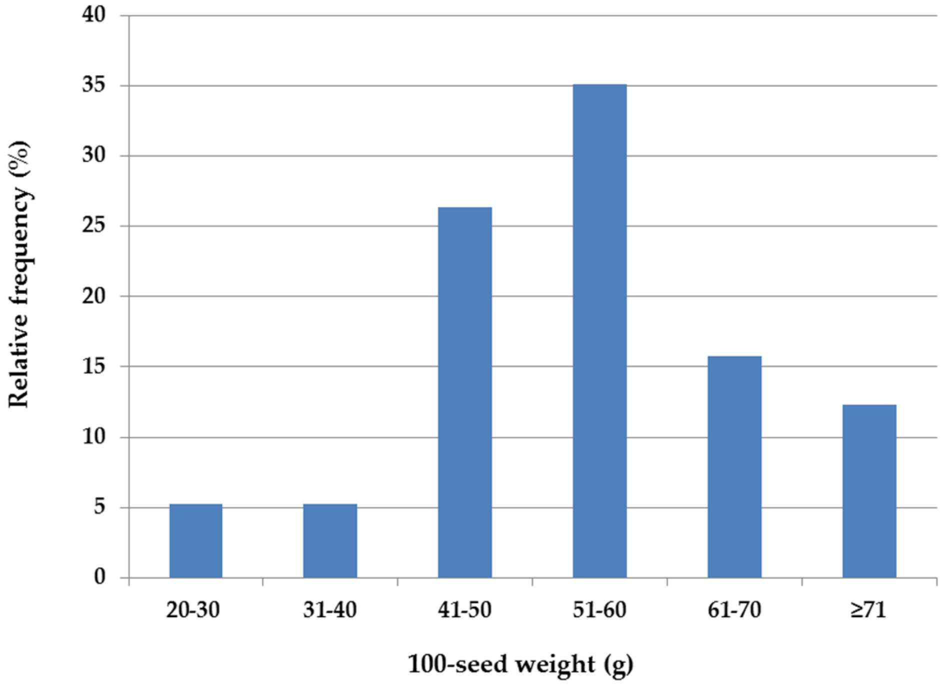

Figure S3. Frequency distribution of six classes of 100-seed weight (g) recorded within the 57 Sicilian common bean landrace.

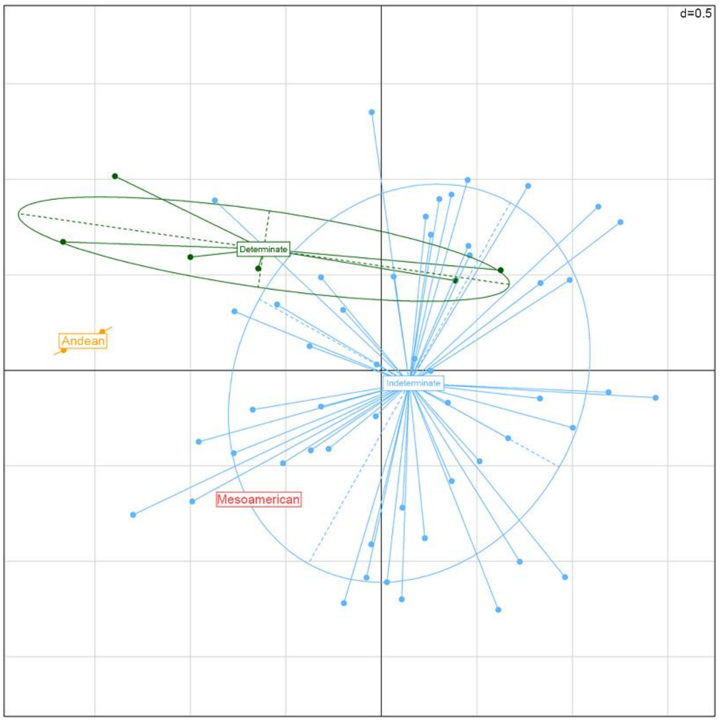

Figure S4. Relationships between determinate and indeterminate Sicilian accessions and Andean (Jalo EEP558, Midas) and Mesoamerica (BAT93) references as represented by the first two principal coordinates of a Principal Coordinates Analysis (PCoA) using allelic profiles from 8 SSR molecular markers.
